# Inpatient Outcomes for Myocarditis-Related Heart Failure

**DOI:** 10.1055/s-0043-1776141

**Published:** 2023-11-03

**Authors:** Mohammad Alabbas, Cheryl Gibson, Abdulrahman Morad, Mohammad Alhoda Mohammad Alahmad

**Affiliations:** 1 Internal Medicine, University of Debrecen, Debrecen, Hungary; 2 Internal Medicine, University of Kansas Medical Center, Kansas City, Kansas, United States; 3 Cardiovascular Medicine, University of Kansas Medical Center, Kansas City, Kansas, United States

**Keywords:** heart failure, inpatient mortality, myocarditis

## Abstract

**Background** Heart failure (HF) is one of the leading causes of hospitalizations among adults, accounting for high rates of morbidity and mortality in the United States. Myocarditis is a less common etiology of HF, and its outcomes are less well understood.

**Methods** We used the Nationwide Readmissions Database from 2016 to 2019, extracting adult patients with a primary diagnosis of HF who were admitted between January and November of each year studied. We excluded patients with missing data on event time or length of stay. Inpatient outcomes were compared between cases of HF without myocarditis and myocarditis-associated HF (MAHF). Survey procedures were applied. Propensity scores as covariates were used in survey-weighted models to estimate the population average treatment effect on the treated using SAS 9.4.

**Results** We included 4,454,272 HF-related weighted admissions for which 4,605 patients (0.1%) had a concurrent diagnosis of myocarditis. Overall, patients with MAHF, compared with HF without myocarditis, were younger (mean age: 53 years vs. 72 years, *p* < 0.001) with fewer women (45 vs. 48%), respectively. Patients with MAHF had more inpatient complications including cardiac arrest, cardiogenic shock, and use of mechanical circulatory support ( *p* < 0.001) despite having fewer comorbidities such as diabetes, hypertension, and renal disease. Patients with MAHF had longer mean lengths of stay (9.2 vs. 5.5 days, *p* < 0.001). In-hospital mortality during index admission was significantly higher in MAHF at 3.9% compared with 2.8% for HF without myocarditis ( *p* < 0.001). Myocarditis was a key predictor of inpatient mortality adjusting for risk factors.

**Conclusion** Myocarditis-related HF is associated with increased inpatient mortality, resource utilization, and prolonged hospitalization.

## Introduction

Myocarditis is defined as inflammation of the myocardial tissue, often resulting from infectious causes. It has also been named inflammatory cardiomyopathy, a leading cause of hospitalizations among elderly adults. It is uncommon, with an estimated incidence between 10 and 22 cases per 100,000 persons. However, it contributes to high rates of cardiovascular morbidity and mortality in the United States. 1 It is often an underdiagnosed cause of heart failure (HF) with little research on the presence and pathological features of myocarditis in the advanced HF population. 2 Clinical features of myocarditis are diverse and overlap with other acute cardiac conditions which makes diagnosis challenging.

Myocarditis may present with a wide range of symptoms. While majority (up to 70%) of cases follow a benign course, myocarditis can also lead to dilated cardiomyopathy and HF. 3 The inflammation predominantly results from a narrow spectrum of viral infections or autoimmune etiologies such as systemic lupus erythematosus. 4 Myocarditis may also develop as a hypersensitivity reaction to medications including penicillins, sulfonamides, and methyldopa. 5 This insult to the myocardium may impair cardiac function. 6 Despite extensive workup, no specific etiology can be identified in up to 30% of biopsy-confirmed cases. 7 The prognosis and treatment of myocarditis, however, varies according to the cause. Timely recognition and treatment of myocarditis is key to preventing poor outcomes. 8

Although the prognosis of myocarditis and the underlying cause and severity of presenting symptoms vary widely, previous studies have found that the presence of myocarditis along with HF has worse outcomes in terms of mortality, inpatient complications, and re-admissions. 9 10 11 12 Such patients have around 28% risks of mortality or heart transplant at 2 months. 13 Patients may also present with nonspecific symptoms like fever, fatigue, chest pain, or palpitations. ^3^ HF symptoms, like dyspnea and edema, are more concerning and depict impaired ventricular function. Sudden cardiogenic shock can occur with fulminant myocarditis. Since clinical features are variable, diagnosis relies on suspicion plus cardiac biomarkers, electrocardiography, imaging, and sometimes biopsy. 6 Cardiac troponins are sensitive indicators of myocardial injury that are usually elevated in myocarditis. 14 Electrocardiography frequently shows nondiagnostic ST and T wave changes along with arrhythmias. 15 Echocardiography identifies wall motion abnormalities and ventricular dysfunction. However, cardiac magnetic resonance imaging (MRI) is superior for visualizing myocardial inflammation and scarring. 6 Endomyocardial biopsy is the gold standard but is reserved for selected cases given its invasive risks. 16 However, the data are sparse, and very little research has been conducted to assess the adverse outcomes of myocarditis associated with HF at a national level. Further, most of the studies related to the clinical outcomes of myocarditis with HF have been conducted in the pediatric population. 17 18 19 The aim of this study, therefore, is to investigate the inpatient outcomes in adult patients admitted with the primary diagnosis of HF with myocarditis compared with those without myocarditis.

## Materials and Methods

Community hospitals represent the majority (85%) of hospitals in the United States. 20 For every hospital encounter, a hospital billing record is created at the time of discharge. These data are sent to authority-related health organizations in each state. From these data, the state inpatient database is created by the Agency for Healthcare Research and Quality through the Healthcare Cost and Utilization Project (HCUP). The Nationwide Readmissions Database (NRD) is created from these data. Approximately 60% of the states in the United States are participating in NRD. 21 The HCUP team takes responsibility to validate the data, clean them up, and make them available for public use to promote research and improve outcomes. Because of strict privacy rules, patients are not identified. Hence, this study was waived by the institutional review board at our institution.

The NRD was created to have nationally representative information on hospital readmissions for all ages. It has weight, cluster, and stratum variables (DISCWT, HOSP_NRD, and NRD_STRATUM), which make it possible to acquire national estimates accurately. The data also have a specific patient key (KEY_NRD), which allows us to track readmission within the same state in any given year. The database includes demographic information such as age and gender. However, it does not include the race variable for privacy purposes. Also, it includes codes that summarize up to 40 clinical diagnoses as well as up to 30 inpatient procedures based on the International Classification of Diseases, 10th revision (ICD 10 codes). Unfortunately, the database does not include laboratory results or imaging reports including echocardiogram reports.

In our study, we included all patients with a primary diagnosis of HF who were discharged between January and November each year, from 2016 to 2019. We did not include the year 2020 because results could have been affected by the coronavirus disease pandemic of 2019. Also, we did not include discharges in December of each year studied to evaluate 30-day readmission.

The codes used to include patients with a primary diagnosis (I10_DX1) of HF have been validated by the HCUP team. 22 Although some ICD-10 codes can specify patients with systolic versus nonsystolic HF, it may not be valid to rely on them.

Unfortunately, there is no specific code for myocarditis-related HF. Hence, we defined a case of myocarditis-related HF if the patient has a diagnostic code of myocarditis with a primary diagnosis of HF (see Supplementary Tables S1 – S3 , available in the online version). Because of data limitations, we are not able to classify the etiology or the chronicity of myocarditis.

We followed the instructions indicated by the HCUP team in the data user agreement. Domain analyses were utilized. 23 Survey procedures were applied to accommodate the complex sampling design (STRATA= NRD_STRATUM, YEAR; CLUSTER= HOSP_NRD; WEIGHT= DISCWT). Discrete variables were reported as percentages. Continuous variables were reported as median with interquartile range or mean with standard deviation. Univariate analyses were performed using the Chi-square and least-squares means tests 24 for discrete and continuous variables, respectively. Because of the complex design, classic methods to obtain propensity scores were avoided. 25 Propensity scores were used as covariates in survey-weighted models to estimate the population average treatment effect on the treated adjusting for age, gender, socioeconomic status, Elixhauser mortality index, and discharge disposition (using PROC PSMATCH procedure, the PSMODEL statements contain myocard(TREATED = '1') = FEMALE AGE CMR_INDEX_MORTALITY ZIPINC_QRTL PL_NCHS DISCWT;). 25 Logistic regression was performed in multivariate analysis. The *p* -value for all analyses was assumed to be 0.05. We used SAS 9.4 for data exploration and analysis.

## Results

**Fig. 1:**
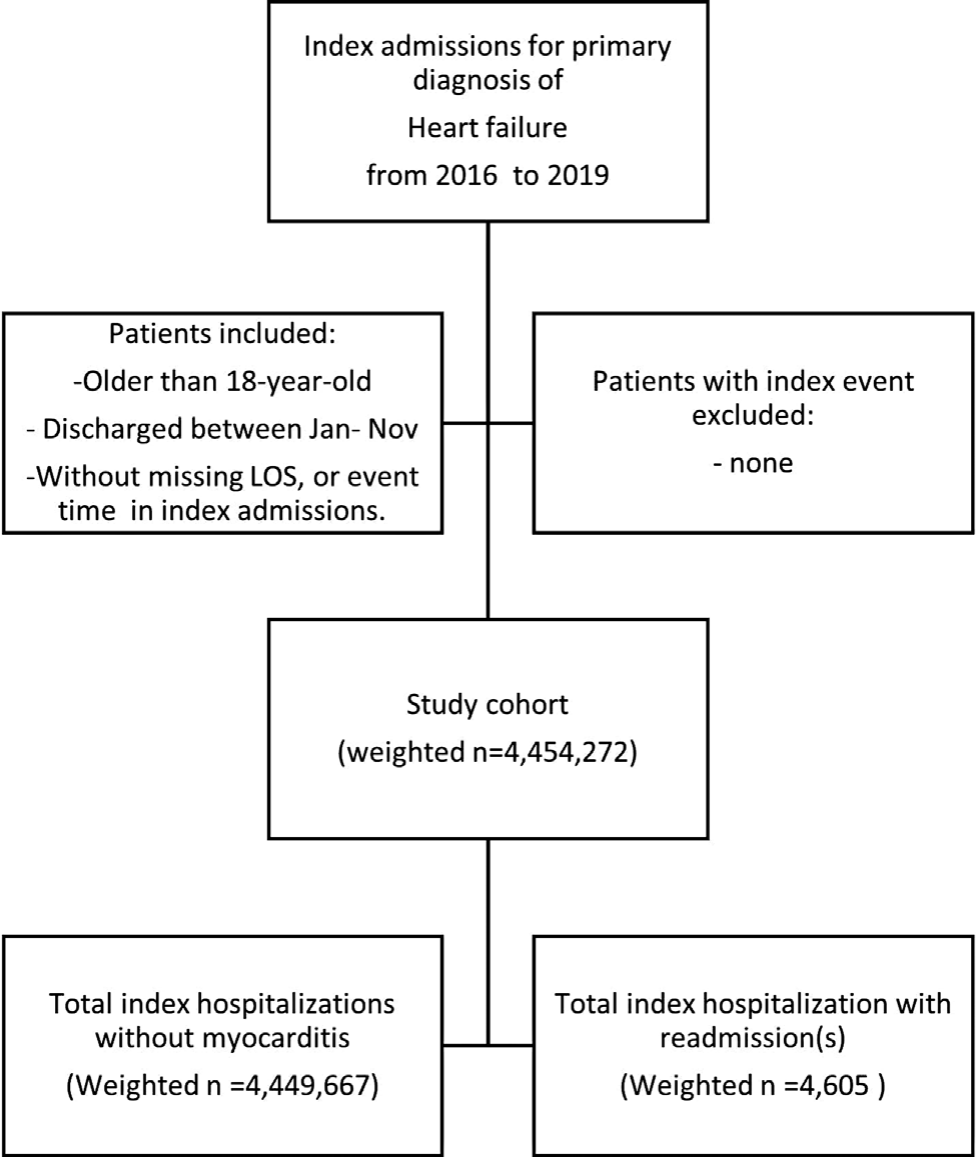
Study flowchart.

In the current study, we identified 4,454,272 weighted hospitalizations between January and November from 2016 to 2019, with a primary discharge diagnosis of HF. Of the total index admissions, 4,605 patients were found to have HF with myocarditis (0.1%), while HF patients without myocarditis accounted for 4,449,667 weighted hospitalizations (99.9%, as shown in Fig. 1 ).

Table 1 demonstrates the baseline demographic and clinical characteristics of the study population, categorized into two groups, i.e., HF with myocarditis and HF without myocarditis. The group of patients having HF with myocarditis was relatively younger (median age: 54.4 vs. 74, *p* -value < 0.001) and had a lesser proportion of women (44.7 vs. 48.1%, *p* -value = 0.003). HF patients without myocarditis had a greater prevalence of most of the co-morbidities, while alcohol consumption, drug abuse, autoimmune diseases, coagulopathy, chronic liver disease, pulmonary hypertension, and peptic ulcer disease were more prevalent in HF patients with myocarditis.

**Table 1:** Baseline demographic and clinical characteristics of the study population

Variable	Total ( *n* = 4,454,272)	With myocarditis ( *n* = 4,605)	Without myocarditis ( *n* = 4,449,667)	*p* -Value
Age median [25th–75th percentile], y	73 [62–83]	54 [41–64]	74 [62–83]	<0.001
Female gender, *n* (%)	2,143,884 (48.1%)	2,058 (44.7%)	2,141,826 (48.1%)	0.0031
Cormorbidities, *n* (%)				
AIDS, *n* (%)	20,560 (0.5%)	20 (0.4%)	20,540 (0.5%)	0.8884
Alcohol abuse, *n* (%)	151,192 (3.4%)	243 (5.3%)	150,949 (3.4%)	<0.0001
Autoimmune disease, *n* (%)	160,790 (3.6%)	314 (6.8%)	160,476 (3.6%)	<0.0001
Chronic lung disease, *n* (%)	1,773,516 (39.8%)	1,052 (22.8%)	1,772,464 (39.8%)	<0.0001
Dementia, *n* (%)	367,719 (8.3%)	38 (0.8%)	367,681 (8.3%)	<0.0001
Depression, *n* (%)	530,532 (11.9%)	483 (10.5%)	530,049 (11.9%)	0.0374
Diabetes mellitus, *n* (%)	2,177,548 (48.9%)	1,293 (28.1%)	2,176,255 (48.9%)	<0.0001
Drug abuse, *n* (%)	172,828 (3.9%)	232 (5.0%)	172,596 (3.9%)	0.007
Hypertension, *n* (%)	1,016,850 (22.8%)	936 (20.3%)	1,015,914 (22.8%)	0.0186
Hypothyroidism, *n* (%)	815,660 (18.3%)	515 (11.2%)	815,146 (18.3%)	<0.0001
Malignancy, *n* (%)	226,683 (5.1%)	180 (3.9%)	226,502 (5.1%)	0.017
Obesity, *n* (%)	1,173,915 (26.4%)	1,217 (26.4%)	1,172,698 (26.4%)	0.9457
PVD, *n* (%)	496,313 (11.1%)	236 (5.1%)	496,077 (11.1%)	<0.0001
Deficiency anemia, *n* (%)	1,464,047 (32.9%)	912 (19.8%)	1,463,135 (32.9%)	<0.0001
Blood loss, *n* (%)	41,315 (0.9%)	31 (0.7%)	41,283 (0.9%)	0.1906
Coagulopathy, *n* (%)	347,419 (7.8%)	575 (12.5%)	346,844 (7.8%)	<0.0001
Chronic liver disease, *n* (%)	306,996 (6.9%)	556 (12.1%)	306,439 (6.9%)	<0.0001
Encephalopathies, *n* (%)	220,306 (4.9%)	179 (3.9%)	220,126 (4.9%)	0.0195
Pulmonary HTN, *n* (%)	1,017,876 (22.9%)	1,202 (26.1%)	1,016,675 (22.8%)	0.002
CKD, *n* (%)	2,258,725 (50.7%)	1,215 (26.4%)	2,257,509 (50.7%)	<0.0001
PUD, *n* (%)	32,952 (0.7%)	57 (1.2%)	32,895 (0.7%)	0.0095
Weight loss, *n* (%)	275,990 (6.2%)	308 (6.7%)	275,683 (6.2%)	0.363
Valvular disease, *n* (%)	1,315,782 (29.5%)	1,320 (28.7%)	1,314,463 (29.5%)	0.3629
Hospital location, *n* (%)				
Central metropolitan, *n* (%)	1,133,911 (25.5%)	1,472 (32.0%)	1,132,439 (25.4%)	<0.0001
Fringe metropolitan, *n* (%)	1,132,177 (25.4%)	1,301 (28.3%)	1,130,876 (25.4%)	
Medium metropolitan, *n* (%)	945,175 (21.2%)	783 (17.0%)	944,392 (21.2%)	
Small metropolitan, *n* (%)	439,147 (9.9%)	407 (8.8%)	438,740 (9.9%)	
Micropolitan counties, *n* (%)	427,552 (9.6%)	349 (7.6%)	427,203 (9.6%)	
Socioeconomic status				
Low, *n* (%)	1,502,845 (33.7%)	1,345 (29.2%)	1,501,500 (33.7%)	<0.0001
Median, *n* (%)	1,204,566 (27.0%)	1,164 (25.3%)	1,203,402 (27.0%)	
50th–75th percentile, *n* (%)	995,513 (22.3%)	1,099 (23.9%)	994,414 (22.3%)	
75th–100th percentile, *n* (%)	695,047 (15.6%)	926 (20.1%)	694,121 (15.6%)	

Abbreviations: AIDS, acquired immunodeficiency virus; CKD, chronic kidney disease; HTN, hypertension; *n* , number; PUD, peptic ulcer disease; PVD, peripheral vascular disease.

Most of the admissions took place in hospitals in large metropolitan areas. Medicare was the primary insurance in a group of HF patients without myocarditis (75%, *p* -value < 0.001), while HF patients with myocarditis used both private and Medicare insurance plans (36.5 and 34.9%, respectively, *p* -value < 0.001).

Table 2 enumerates the outcomes of the primary study. The mortality during index admission was significantly higher in the myocarditis group (3.9 vs. 2.8%, *p* = 0.0034). Similarly, these patients had a longer length of stay (LOS; median: 5 days vs. 4 days, *p* -value < 0.001) and more median hospital charges ($55,049 vs. $30,783, *p* -value < 0.001) compared with the HF patients without myocarditis. Moreover, there was a higher prevalence of inpatient complications, including ventricular fibrillation, cardiogenic shock, and cardiac arrest in HF patients with myocarditis. Despite their younger age and relatively healthier profile, over a third of patients with myocarditis-associated HF (MAHF) were not ready to be discharged home by the end of their index hospitalization.

**Table 2:** Primary outcomes

Variable	Total *n* = 4,454,272	With myocarditis ( *n* = 4,605)	Without myocarditis ( *n* = 4,449,667)	*p* -Value
Index mortality, *n* (%)	125,872 (2.8%)	179 (3.9%)	125,693 (2.8%)	0.0034
LOS median [25th–75th percentile], d	4 [2–7]	5 [3–10]	4 [2–7]	<0.001
Total charges median [25th–75th percentile] in U.S. dollar	30,798 [17,823–55,876]	55,049 [28,263–119,655]	30,783 [17,817–55,834]	<0.0001
Cardiogenic shock, *n* (%)	101,501 (2.3%)	867 (18.8%)	100634 (2.3%)	<0.0001
Total SCA, *n* (%)	41,946 (0.9%)	171 (3.7%)	41,775 (0.9%)	<0.0001
Not procedure-related arrest, *n* (%)	30,938 (0.7%)	110 (2.4%)	30,828 (0.7%)	<0.0001
Procedure-related arrest, *n* (%)	1,222 (0.0%)	11 (0.2%)	1,211 (0.0%)	<0.0001
VF, *n* (%)	14,172 (0.3%)	78 (1.7%)	14,093 (0.3%)	<0.0001
Discharge disposition				
Discharged home, *n* (%)	2,238,753 (50.3%)	3,054 (66.3%)	2,235,699 (50.2%)	<0.0001
Transfer to short-term, *n* (%)	44,964 (1.0%)	151 (3.3%)	44,812 (1.0%)	
Discharged to a facility, *n* (%)	822,523 (18.5%)	254 (5.5%)	822,269 (18.5%)	
Home health care, *n* (%)	1,149,417 (25.8%)	895 (19.4%)	1,148,523 (25.8%)	
30-day readmission, *n* (%)	1,013,525 (23.4%)	867 (19.5%)	1,012,658 (23.4%)	<0.0001
Cardiac MRI, *n* (%)	483 (0.0%)	18 (0.4%)	464 (0.0%)	<0.0001
Right HC, *n* (%)	102,491 (2.3%)	787 (17.1%)	101,704 (2.3%)	<0.0001
Left HC, *n* (%)	207,258 (4.7%)	861 (18.7%)	206,397 (4.6%)	<0.0001
Combined HC, *n* (%)	108,842 (2.4%)	597 (13.0%)	108,246 (2.4%)	<0.0001
IABP, *n* (%)	12,562 (0.3%)	202 (4.4%)	12,360 (0.3%)	<0.0001
VA-ECMO, *n* (%)	2,513 (0.1%)	102 (2.2%)	2,411 (0.1%)	<0.0001
PVAD, *n* (%)	11,087 (0.2%)	174 (3.8%)	10,913 (0.2%)	<0.0001
LVAD, *n* (%)	9,618 (0.2%)	123 (2.7%)	9,496 (0.2%)	<0.0001
Heart transplant, *n* (%)	5,109 (0.1%)	86 (1.9%)	5,023 (0.1%)	<0.0001
Days to readmission median [25th–75th percentile], d	12 [6–20]	11 [6–19]	12 [6–20]	0.0851
First readmission mortality, *n* (%)	63,056 (1.4%)	45 (5%)	63,011 (6%)	0.3677
Readmission LOS median [25th–75th percentile], d	4 [3–8]	5 [3–10]	4 [3–8]	<0.001

Abbreviations: CABG, coronary artery bypass grafting; HC, heart catheterization; IABP, intra-aortic balloon pump; LOS, length of stay; LVAD, left ventricular assist device; MRI, magnetic resonance imaging; *n* , number; PVAD, percutaneous ventricular assist device; SCA, sudden cardiac arrest; VA-ECMO, veno-arterial extracorporeal membrane oxygenation; VF, ventricular fibrillation.

The all-cause 30-day readmission rate was up to 19% in HF patients with myocarditis, with a similar average number of days before readmission and a much longer LOS during the first readmission after the index hospitalization.

Data showed that HF patients with myocarditis availed themselves of more inpatient resources, including cardiac MRI, heart catheterizations, and advanced HF therapy.

## Propensity Score Analysis

**Fig. 2:**
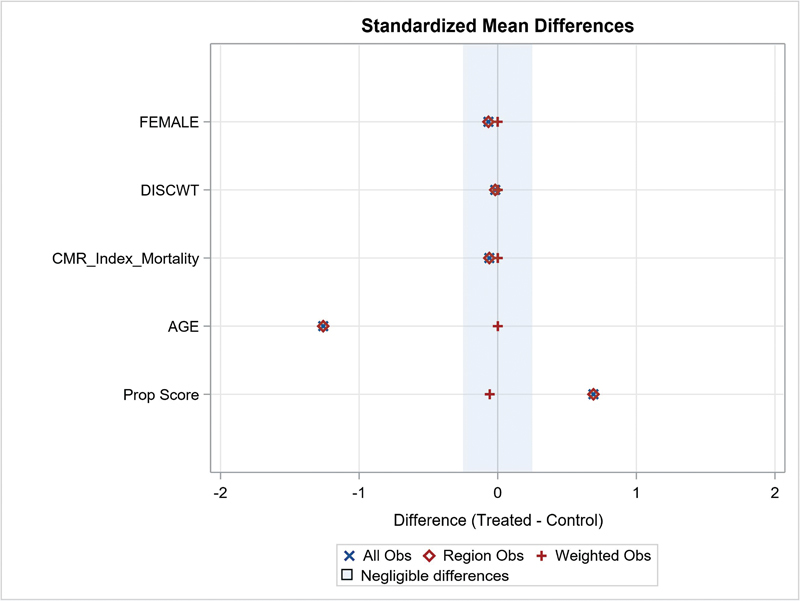
Assessment of propensity score analysis.

An assessment of propensity score analysis (PSA) was done as shown in Fig. 2 . Considerable standardized mean differences were present before to analyses.

Tables 3 and 4 demonstrate basic characteristics and inpatient outcomes after applying PSA. They show that HF patients with myocarditis were relatively younger than the patients without myocarditis. However, no statistically significant difference was found in the female preponderance between the two groups. HF patients with myocarditis had significantly higher inpatient mortality during index admission (3.9 vs. 2.3%, *p* < 0.0001), as well as a higher prevalence of inpatient complications and higher utilization of inpatient resources. However, no statistical significance was found between the two groups in inpatient mortality rate during the first readmission. The lower 30-day readmission rate for the group with myocarditis could be at least partially explained by higher mortality rates during the index admission.

**Table 3:** Basic characteristic after applying propensity score analysis

Variable	With myocarditis	Without myocarditis	*p* -Value
Total	4,527	4,526	
Age means (SD), y	52.4 (21.7)	52.4 (0.7)	<0.001
Female gender, *n* (%)	2,020 (44.6%)	2,042 (45.1%)	0.6934
AIDS, *n* (%)	20 (0.4%)	47 (1.0%)	0.0116
Alcohol abuse, *n* (%)	236 (5.2%)	264 (5.8%)	0.2647
Autoimmune disease, *n* (%)	311 (6.9%)	183 (4.0%)	<0.0001
Chronic lung disease, *n* (%)	1,026 (22.7%)	1,520 (33.6%)	<0.0001
Dementia, *n* (%)	38 (0.8%)	116 (2.6%)	<0.0001
Depression, *n* (%)	481 (10.6%)	517 (11.4%)	0.2365
Diabetes mellitus, *n* (%)	1,268 (28.0%)	2,007 (44.3%)	<0.0001
Drug abuse, *n* (%)	224 (4.9%)	397 (8.8%)	<0.0001
Hypertension, *n* (%)	920 (20.3%)	1,119 (24.7%)	<0.0001
Hypothyroidism, *n* (%)	508 (11.2%)	520 (11.5%)	0.7184
Malignancy, *n* (%)	178 (3.9%)	174 (3.8%)	0.8048
Obesity, *n* (%)	1,208 (26.7%)	1,574 (34.8%)	<0.0001
PVD, *n* (%)	235 (5.2%)	339 (7.5%)	<0.0001
Deficiency anemia, *n* (%)	899 (19.9%)	1,461 (32.3%)	<0.0001
Coagulopathy, *n* (%)	567 (12.5%)	435 (9.6%)	<0.0001
Chronic liver disease, *n* (%)	547 (12.1%)	509 (11.2%)	0.2302
Encephalopathies, *n* (%)	179 (4.0%)	239 (5.3%)	0.0048
Pulmonary HTN, *n* (%)	1,187 (26.2%)	1,059 (23.4%)	0.007
CKD, *n* (%)	1,198 (26.5%)	2,063 (45.6%)	<0.0001
Weight loss, *n* (%)	304 (6.7%)	302 (6.7%)	0.9233
Valvular disease, *n* (%)	1,301 (28.7%)	1,102 (24.3%)	<0.0001
Hospital location			
Central metropolitan, *n* (%)	1,457 (32.2%)	1,420 (31.4%)	0.8884
Fringe metropolitan, *n* (%)	1,297 (28.7%)	1,337 (29.5%)	
Medium, *n* (%) metropolitan	771 (17.0%)	792 (17.5%)	
Small metropolitan, *n* (%)	402 (8.9%)	388 (8.6%)		
Micropolitan counties, *n* (%)	328 (7.3%)	339 (7.5%)		
Socioeconomic status			
Low, *n* (%)	1,340 (29.6%)	1,344 (29.7%)	0.9974
Median, *n* (%)	1,162 (25.7%)	1,154 (25.5%)	
50–75 percentile, *n* (%)	1,097 (24.2%)	1,096 (24.2%)	
75–100 percentile, *n* (%)	926 (20.5%)	932 (20.6%)	

Abbreviations: AIDS, acquired immunodeficiency virus; CKD, chronic kidney disease; HTN, hypertension; n, number; PUD, Peptic ulcer disease; PVD, Peripheral vascular disease; SD, standard deviation.

**Table 4:** Outcome table after applying propensity score analysis

Outcome	With myocarditis	Without myocarditis	*p* -Value
Total, *n*	4,526	4,527	
Index mortality, *n* (%)	176 (3.9%)	103 (2.3%)	<0.0001
LOS mean (SD), days	9.2 (18.3)	6.2 (0.4)	<0.0001
Total charges mean (SD), in U.S. dollar	147,381.7 (453,054.5)	75,955.6 (9,737.0)	<0.0001
Cardiogenic shock, *n* (%)	851 (18.8%)	213 (4.7%)	<0.0001
Overall SCA, *n* (%)	167 (3.7%)	60 (1.3%)	<0.0001
Not procedure-related arrest, *n* (%)	107 (2.4%)	40 (0.9%)	<0.0001
Procedure-related arrest, *n* (%)	11 (0.2%)	3 (0.1%)	0.0026
VF, *n* (%)	77 (1.7%)	25 (0.6%)	<0.0001
Discharge disposition			
Discharged Home, *n* (%)	2,993 (66.1%)	2,989 (66.0%)	0.9974
Transfer to Short-term, *n* (%)	148 (3.3%)	59 (1.3%)	
Discharged to a facility, *n* (%)	254 (5.6%)	411 (9.1%)	
Home health Care, *n* (%)	886 (19.6%)	815 (18.0%)	
30-day readmission, *n* (%)	855 (19.6%)	1,129 (25.5%)	<0.0001
Cardiac MRI, *n* (%)	18 (0.4%)	1 (0.0%)	<0.0001
Right HC, *n* (%)	776 (17.1%)	219 (4.8%)	<0.0001
Left HC, *n* (%)	841 (18.6%)	267 (5.9%)	<0.0001
Combined HC, *n* (%)	589 (13.0%)	164 (3.6%)	<0.0001
IABP, *n* (%)	200 (4.4%)	36 (0.8%)	<0.0001
VA-ECMO, *n* (%)	102 (2.3%)	14 (0.3%)	<0.0001
PVAD, *n* (%)	170 (3.8%)	32 (0.7%)	<0.0001
LVAD, *n* (%)	121 (2.7%)	35 (0.8%)	<0.0001
Heart transplant, *n* (%)	83 (1.8%)	26 (0.6%)	<0.0001
Days to readmission mean (SD), days	12.6 (11.3)	13.1 (0.4)	0.1819
First readmission mortality, *n* (%)	45 (5%)	49 (4.3%)	0.3402
Readmission LOS mean (SD), days	9.5 (18.9)	6.8 (0.4)	<0.0001

Abbreviations: CABG, coronary artery bypass grafting; HC, heart catheterization; IABP, intra-aortic balloon pump; LOS, length of stay; LVAD, left ventricular assist device; MRI, magnetic resonance imaging; *n* , number; PVAD, percutaneous ventricular assist device; SCA, sudden cardiac arrest; SD, standard deviation; VA-ECMO, veno-arterial extracorporeal membrane oxygenation; VF, ventricular fibrillation.

**Fig. 3:**
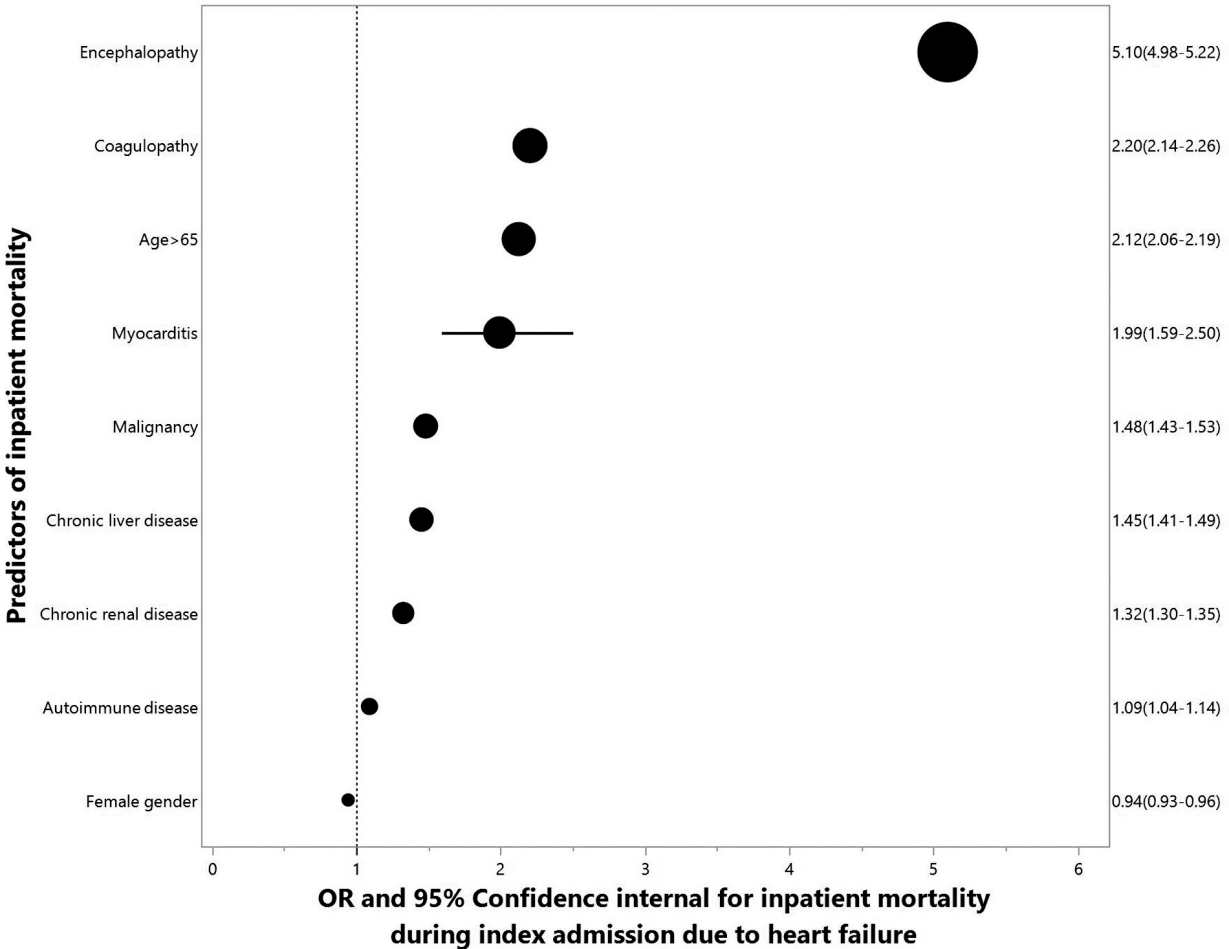
Results of multivariate analysis to evaluate contributing factors to inpatient mortality.

Fig. 3 displays the odds ratio for inpatient mortality during index hospitalizations for patients admitted with a primary diagnosis of HF. After adjustment for age, gender, and other comorbidities, HF patients with myocarditis had two times the odds of inpatient mortality than the HF patients without myocarditis (95% confidence interval: 1.6–2.5, *p* -value < 0.001).

## Discussion

Myocarditis, a relatively infrequent cardiac condition, constitutes a mere 0.1% of HF admissions within our studied population. However, its clinical significance becomes evident through the heightened rates of adverse in-hospital events, positioning myocarditis as a pivotal prognostic determinant in HF patients. Our current investigation not only reaffirms prior research indicating heightened morbidity and mortality in cases of MAHF, but it also expands our understanding of this relationship.

## Clinical Outcomes and Mortality

Our findings illustrate a higher in-hospital mortality rates among MAHF patients, standing at 3.9% in contrast to the 2.8% observed in HF cases devoid of myocarditis. This absolute risk increase of 1.1% translates into a 39% relative escalation in mortality risk. This observation aligns seamlessly with findings from an extensive Danish study where 90-day mortality rates reached 4.9% for myocarditis patients compared with 3.4% in population controls. 12 Furthermore, our longitudinal data underscore this phenomenon, revealing a 30-day readmission mortality rate of 5% in MAHF patients versus 4.3% in the control group. The escalated mortality rates are largely attributable to the increased incidence of in-hospital complications, including ventricular fibrillation, cardiac arrest, and cardiogenic shock, specifically within the MAHF cohort.

## Resource Utilization and Length of Stay

It is notable that the median duration of hospitalization for MAHF patients exceeded that of their counterparts by a day, accompanied by a considerable surge in median hospital charges, surpassing an increment of $20,000. This observation coheres with earlier data derived from cohorts afflicted by viral myocarditis, where prolonged periods of intensive care and overall hospitalization were demonstrated. 9 This augmented resource allocation is demonstrative of the increased severity of illness within the MAHF patient population, necessitating heightened monitoring, interventional procedures, and specialized care.

## Independent Impact of Myocarditis

Notably, despite the comparatively younger average age and a lower prevalence of comorbidities such as diabetes and chronic kidney disease, the MAHF patients exhibited inferior clinical outcomes. Following meticulous adjustment for potential confounders, it becomes evident that myocarditis independently confers a twofold elevation in the odds of in-hospital mortality. This distinct association underscores myocarditis itself as the primary contributor to unfavorable outcomes, surpassing demographic or clinical factors in significance.

## Diagnostic Approaches and Interventions

Diagnostic strategies employed within the study delineate a propensity for cardiac MRI and invasive angiography in MAHF patients, echoing established guidelines that endorse cardiac MRI as the modality of choice for suspected myocarditis. 6 Furthermore, the role of angiography in ruling out ischemic etiologies of cardiomyopathy is accentuated. 16 The augmented frequency of advanced HF interventions, encompassing ventricular assist devices and transplantation, serves as a reflection of the severity of illness within this particular cohort.

## Long-Term Prognosis and Pediatric Correlation

Existing literature has elucidated varying long-term prognoses for myocarditis patients, ranging from complete recovery to augmented risks of dilated cardiomyopathy, sudden cardiac demise, and recurrent myocarditis. In line with this, our study underscores that MAHF patients continue to manifest inferior outcomes following discharge, with heightened readmission rates and postdischarge mortality in comparison to HF patients without myocarditis. Worth noting, analogous trends have been observed in pediatric studies, which depict amplified complications, therapeutic interventions, and mortality rates in cases of myocarditis. 26 27 28 29

## Research Gaps and Study Limitations

While pediatric populations have garnered relatively more attention in the literature, a paucity of evidence persists regarding outcomes in adult MAHF patients. An early study by Grogan et al in 1995 reported no significant discrepancy in 5-year survival between biopsy-confirmed MAHF patients and matched dilated cardiomyopathy controls. 17 18 19 However, our contemporary investigation augments the existing literature with robust data on in-hospital morbidity and mortality associated with MAHF.

This study has some limitations worth noting. First, as an administrative database, the NRD lacks vital signs, ethnicity, anthropometric measurements, laboratory results, pathology reports, and ejection fraction. Second, the retrospective design may introduce bias. Furthermore, case definition is according to ICD-10 codes which predisposes to limitation including coding errors. We were unable to determine myocarditis acuity, severity, or etiology. Misclassification is also possible given the lack of chart review. The restricted timing also precluded analyzing longer term prognosis (NRD does not allow tracking a de-identified patient across states or across years). Further studies are warranted focusing on postdischarge outcomes. Validation and prospective studies are needed.

## Knowledge Gaps

- Limited evidence on in-hospital outcomes of adult myocarditis-associated heart failure (MAHF) patients compared with heart failure alone.
- Prior studies had small sample sizes or were mostly focused on pediatric populations.
- Lacked contemporary national data on morbidity, mortality, and health care utilization in MAHF.

## Key Findings

- MAHF patients had higher in-hospital mortality compared with heart failure alone (3.9 vs. 2.8%).
- MAHF associated with slightly longer hospital stays and higher hospitalization charges.
- MAHF patients had more in-hospital complications like cardiac arrest despite younger age and fewer comorbidities.
- Myocarditis was an independent predictor of in-hospital mortality after adjusting for confounders.

## Contributions

- Provides contemporary national data on 4.6 million heart failure hospitalizations including over 4,600 with myocarditis.
- Represents the largest study to date focused on inpatient outcomes of adult MAHF patients.
- Addresses gap in literature regarding morbidity, mortality, and health care utilization in this population.
- Highlights myocarditis as a major risk factor for poor in-hospital outcomes independent of demographics and comorbidities.
- Underscores the need for heightened clinical suspicion and aggressive management in MAHF.
- Findings can help guide prognosis, risk stratification, and clinical decision-making for this high-risk group.

Therefore, this nationwide study represents the largest investigation of MAHF outcomes to date. Our findings demonstrate a distinct risk profile in MAHF patients with significantly higher in-hospital mortality, complications, health resource utilization, and 30-day readmission rates. Myocarditis was an independent predictor of poor in-hospital outcomes after adjusting for potential confounders. These results highlight the prognostic importance of myocarditis in HF patients. Further research is needed to clarify optimal management strategies. Nonetheless, this study provides compelling contemporary evidence that the presence of myocarditis in patients requiring hospitalization for decompensated heart failure has a major negative impact across the spectrum of HF-related hospital outcomes.
