# Supplementary material for: Inpatient Outcomes for Myocarditis-Related Heart Failure
Source: Avicenna J Med. 2023 Nov 3;13(4):237–46. doi: 10.1055/s-0043-1776141 (PMC10736203; doi:10.1055/s-0043-1776141)
Supplement: Supplementary file 1 — Supplementary Material [file 10-1055-s-0043-1776141-s2363.pdf]

Supplementary Data

Supplementary Table S1 ICD-10 codes for diagnoses used in study design (International Classification of Diseases, 10th Edition [ICD-10])

	ICD-10 Codes
Heart failure	“I0981,” “I501,” “I5020,” “I5021,” “I5022,” “I5023,” “I5030,” “I5031,” “I5032,” “I5033,” “I5040,” “I5041,” “I5042,” “I5043,” “I50810,” “I50811,” “I50812,” “I50813,” “I50814,” “I5082,” “I5083,” “I5084,” “I5089,” “I509,” “I5181,” “I97130,” “I97131,” “O29121,” “O29122,” “O29123,” “O29129,” “I110,” “I130,” “I132”
Myocarditis	“A381,” ”A3952,” ”B2682,” ”B3322,” ”B5881,” ”D8685,” ”I012,” ”I090,” ”I400,” ”I401,” ”I408,” ”I409,” ”I41,” “I514,” ”J1082,” ”J1182”
Coagulopathy	“D6109,” “D611,” “D612,” “D613,” “D61810,” “D61811,” “D61818,” “D6182,” “D6189,” “D619,” “D65,” “D66,” “D67,” “D680,” “D681,” “D682,” “D68311,” “D68312,” “D68318,” “D6832,” “D684,” “D688,” “D689,” “D691,” “D693,” “D6941,” “D6942,” “D6949,” “D6951,” “D6959,” “D696,” “D698,” “D699,” “D7582,” “O99111,” “O99112,” “O99113,” “O99119,” “O9912,” “O9913”
Chronic liver disease	“A5145,” “A5274,” “B180,” “B181,” “B182,” “B188,” “B189,” “B1910,” “B1920,” “B199,” “B251,” “B581,” “K700,” “K702,” “K7030,” “K7031,” “K709,” “K713,” “K714,” “K7150,” “K7151,” “K716,” “K717,” “K718,” “K730,” “K731,” “K732,” “K738,” “K739,” “K740,” “K7400,” “K7401,” “K7402,” “K741,” “K742,” “K743,” “K744,” “K745,” “K7460,” “K7469,” “K751,” “K752,” “K753,” “K754,” “K7581,” “K7589,” “K759,” “K760,” “K761,” “K762,” “K763,” “K764,” “K7681,” “K7689,” “K769,” “K77,” “B190,” “B1911,” “B1921,” “I8500,” “I8501,” “I8510,” “I8511,” “I864,” “K7040,” “K7041,” “K7210,” “K7211,” “K7290,” “K7291,” “K765,” “K766,” “K767,” “K9182,” “Z944”
Chronic kidney disease	“N184,” “N185,” “N186,” “Z4901,” “Z4902,” “Z4931,” “Z4932,” “Z9115,” “Z940,” “Z992,” “N183,” “N1830,” “N1831,” “N1832,” “N189,” “N19”
Encephalopathy	“E7500,” “E7501,” “E7502,” “E7509,” “E7510,” “E7511,” “E7519,” “E7523,” “E7525,” “E7526,” “E7529,” “E754,” “F05,” “F842,” “G35,” “G360,” “G368,” “G369,” “G370,” “G371,” “G372,” “G373,” “G374,” “G375,” “G378,” “G379,” “G47411,” “G47419,” “G47421,” “G47429,” “G890,” “G910,” “G911,” “G912,” “G913,” “G914,” “G918,” “G919,” “G930,” “G9340,” “G9341,” “G9349,” “G935,” “G936,” “G937,” “G9381,” “G9382,” “G9389,” “G939,” “G94,” “O99350,” “O99351,” “O99352,” “O99353,” “O99354,” “O99355,” “P9160,” “P9161,” “P9162,” “P9163”
Autoimmune	“A1801,” “A1802,” “A3984,” “A5441,” “A5442,” “L4050,” “L4051,” “L4054,” “L4059,” “L900,” “L940,” “L941,” “L943,” “M0500,” “M05011,” “M05012,” “M05019,” “M05021,” “M05022,” “M05029,” “M05031,” “M05032,” “M05039,” “M05041,” “M05042,” “M05049,” “M05051,” “M05052,” “M05059,” “M05061,” “M05062,” “M05069,” “M05071,” “M05072,” “M05079,” “M0509,” “M0510,” “M05111,” “M05112,” “M05119,” “M05121,” “M05122,” “M05129,” “M05131,” “M05132,” “M05139,” “M05141,” “M05142,” “M05149,” “M05151,” “M05152,” “M05159,” “M05161,” “M05162,” “M05169,” “M05171,” “M05172,” “M05179,” “M0519,” “M0520,” “M05211,” “M05212,” “M05219,” “M05221,” “M05222,” “M05229,” “M05231,” “M05232,” “M05239,” “M05241,” “M05242,” “M05249,” “M05251,” “M05252,” “M05259,” “M05261,” “M05262,” “M05269,” “M05271,” “M05272,” “M05279,” “M0529,” “M0530,” “M05311,” “M05312,” “M05319,” “M05321,” “M05322,” “M05329,” “M05331,” “M05332,” “M05339,” “M05341,” “M05342,” “M05349,” “M05351,” “M05352,” “M05359,” “M05361,” “M05362,” “M05369,” “M05371,” “M05372,” “M05379,” “M0539,” “M0540,” “M05411,” “M05412,” “M05419,” “M05421,” “M05422,” “M05429,” “M05431,” “M05432,” “M05439,” “M05441,” “M05442,” “M05449,” “M05451,” “M05452,” “M05459,” “M05461,” “M05462,” “M05469,” “M05471,” “M05472,” “M05479,” “M0549,” “M0550,” “M05511,” “M05512,” “M05519,” “M05521,” “M05522,” “M05529,” “M05531,” “M05532,” “M05539,” “M05541,” “M05542,” “M05549,” “M05551,” “M05552,” “M05559,” “M05561,” “M05562,” “M05569,” “M05571,” “M05572,” “M05579,” “M0559,” “M0560,” “M05611,” “M05612,” “M05619,” “M05621,” “M05622,” “M05629,” “M05631,” “M05632,” “M05639,” “M05641,” “M05642,” “M05649,” “M05651,” “M05652,” “M05659,” “M05661,” “M05662,” “M05669,” “M05671,” “M05672,” “M05679,” “M0569,” “M0570,” “M05711,” “M05712,” “M05719,” “M05721,” “M05722,” “M05729,” “M05731,” “M05732,” “M05739,” “M05741,” “M05742,” “M05749,” “M05751,” “M05752,” “M05759,” “M05761,” “M05762,” “M05769,” “M05771,” “M05772,” “M05779,” “M0579,” “M057A,” “M0580,” “M05811,” “M05812,” “M05819,” “M05821,” “M05822,” “M05829,” “M05831,” “M05832,” “M05839,” “M05841,” “M05842,” “M05849,” “M05851,” “M05852,” “M05859,” “M05861,” “M05862,” “M05869,” “M05871,” “M05872,” “M05879,” “M0589,” “M058A,” “M059,” “M0600,” “M06011,” “M06012,” “M06019,” “M06021,” “M06022,” “M06029,” “M06031,” “M06032,” “M06039,” “M06041,” “M06042,” “M06049,” “M06051,”

(*Continued*)

 © 2023. The Author(s).

Supplementary Table S1 (*Continued*)

	ICD-10 Codes
	"M06052," "M06059," "M06061," "M06062," "M06069," "M06071," "M06072," "M06079," "M0608," "M0609," "M060A," "M061," "M0620," "M06211," "M06212," "M06219," "M06221," "M06222," "M06229," "M06231," "M06232," "M06239," "M06241," "M06242," "M06249," "M06251," "M06252," "M06259," "M06261," "M06262," "M06269," "M06271," "M06272," "M06279," "M0628," "M0629," "M0630," "M06311," "M06312," "M06319," "M06321," "M06322," "M06329," "M06331," "M06332," "M06339," "M06341," "M06342," "M06349," "M06351," "M06352," "M06359," "M06361," "M06362," "M06369," "M06371," "M06372," "M06379," "M0638," "M0639," "M064," "M0680," "M06811," "M06812," "M06819," "M06821," "M06822," "M06829," "M06831," "M06832," "M06839," "M06841," "M06842," "M06849," "M06851," "M06852," "M06859," "M06861," "M06862," "M06869," "M06871," "M06872," "M06879," "M0688," "M0689," "M068A," "M069," "M0760," "M07611," "M07612," "M07619," "M07621," "M07622," "M07629," "M07631," "M07632," "M07639," "M07641," "M07642," "M07649," "M07651," "M07652," "M07659," "M07661," "M07662," "M07669," "M07671," "M07672," "M07679," "M0768," "M0769," "M0800," "M08011," "M08012," "M08019," "M08021," "M08022," "M08029," "M08031," "M08032," "M08039," "M08041," "M08042," "M08049," "M08051," "M08052," "M08059," "M08061," "M08062," "M08069," "M08071," "M08072," "M08079," "M0808," "M0809," "M080A," "M081," "M0820," "M08211," "M08212," "M08219," "M08221," "M08222," "M08229," "M08231," "M08232," "M08239," "M08241," "M08242," "M08249," "M08251," "M08252," "M08259," "M08261," "M08262," "M08269," "M08271," "M08272," "M08279," "M0828," "M0829," "M082A," "M083," "M0840," "M08411," "M08412," "M08419," "M08421," "M08422," "M08429," "M08431," "M08432," "M08439," "M08441," "M08442," "M08449," "M08451," "M08452," "M08459," "M08461," "M08462," "M08469," "M08471," "M08472," "M08479," "M0848," "M084A," "M0880," "M08811," "M08812," "M08819," "M08821," "M08822," "M08829," "M08831," "M08832," "M08839," "M08841," "M08842," "M08849," "M08851," "M08852," "M08859," "M08861," "M08862," "M08869," "M08871," "M08872," "M08879," "M0888," "M0889," "M0890," "M08911," "M08912," "M08919," "M08921," "M08922," "M08929," "M08931," "M08932," "M08939," "M08941," "M08942," "M08949," "M08951," "M08952," "M08959," "M08961," "M08962," "M08969," "M08971," "M08972," "M08979," "M0898," "M0899," "M089A," "M1200," "M12011," "M12012," "M12019," "M12021," "M12022," "M12029," "M12031," "M12032," "M12039," "M12041," "M12042," "M12049," "M12051," "M12052," "M12059," "M12061," "M12062," "M12069," "M12071," "M12072," "M12079," "M1208," "M1209," "M300," "M301," "M302," "M303," "M308," "M310," "M311," "M3110," "M3111," "M3119," "M312," "M3130," "M3131," "M314," "M315," "M316," "M317," "M318," "M319," "M320," "M3210," "M3212," "M3213," "M3214," "M3215," "M3219," "M328," "M329," "M3300," "M3301," "M3302," "M3303," "M3309," "M3310," "M3311," "M3312," "M3313," "M3319," "M3320," "M3321," "M3322," "M3329," "M3390," "M3391," "M3392," "M3393," "M3399," "M340," "M341," "M342," "M3481," "M3482," "M3483," "M3489," "M349," "M3500," "M3501," "M3502," "M3503," "M3504," "M3505," "M3506," "M3507," "M3508," "M3509," "M350A," "M350B," "M350C," "M351," "M352," "M353," "M355," "M356," "M358," "M3581," "M3589," "M359," "M360," "M361," "M368," "M450," "M451," "M452," "M453," "M454," "M455," "M456," "M457," "M458," "M459," "M45A0," "M45A1," "M45A2," "M45A3," "M45A4," "M45A5," "M45A6," "M45A7," "M45A8," "M45AB"
Malignancies	
Metastatic disease	"C770," "C771," "C772," "C773," "C774," "C775," "C778," "C779," "C7800," "C7801," "C7802," "C781," "C782," "C7830," "C7839," "C784," "C785," "C786," "C787," "C7880," "C7889," "C7900," "C7901," "C7902," "C7910," "C7911," "C7919," "C792," "C7931," "C7932," "C7940," "C7949," "C7951," "C7952," "C7960," "C7961," "C7962," "C7970," "C7971," "C7972," "C7981," "C7982," "C7989," "C799," "C7B00," "C7B01," "C7B02," "C7B03," "C7B04," "C7B09," "C7B1," "C7B8," "C800"
Leukemias	"C9010," "C9011," "C9012," "C9100," "C9101," "C9102," "C9110," "C9111," "C9112," "C9130," "C9131," "C9132," "C9140," "C9141," "C9142," "C9150," "C9151," "C9152," "C9160," "C9161," "C9162," "C9190," "C9191," "C9192," "C91A0," "C91A1," "C91A2," "C91Z0," "C91Z1," "C91Z2," "C9200," "C9201," "C9202," "C9210," "C9211," "C9212," "C9220," "C9221," "C9222," "C9230," "C9231," "C9232," "C9240," "C9241," "C9242," "C9250," "C9251," "C9252," "C9260," "C9261," "C9262," "C9290," "C9291," "C9292," "C92A0," "C92A1," "C92A2," "C92Z0," "C92Z1," "C92Z2," "C9300," "C9301," "C9302," "C9310," "C9311," "C9312," "C9330," "C9331," "C9332," "C9390," "C9391," "C9392," "C93Z0," "C93Z1," "C93Z2," "C9400," "C9401," "C9402," "C9420," "C9421," "C9422," "C9430," "C9431," "C9432," "C9440," "C9441," "C9442," "C946," "C9480," "C9481," "C9482," "C9500," "C9501," "C9502," "C9510," "C9511," "C9512," "C9590," "C9591," "C9592"
Lymphoma	"C8100," "C8101," "C8102," "C8103," "C8104," "C8105," "C8106," "C8107," "C8108," "C8109," "C8110," "C8111," "C8112," "C8113," "C8114," "C8115," "C8116," "C8117," "C8118," "C8119," "C8120," "C8121," "C8122," "C8123," "C8124," "C8125," "C8126," "C8127," "C8128," "C8129,"

 © 2023. The Author(s).

Supplementary Table S1 (*Continued*)

	ICD-10 Codes
	"C8130," "C8131," "C8132," "C8133," "C8134," "C8135," "C8136," "C8137," "C8138," "C8139," "C8140," "C8141," "C8142," "C8143," "C8144," "C8145," "C8146," "C8147," "C8148," "C8149," "C8170," "C8171," "C8172," "C8173," "C8174," "C8175," "C8176," "C8177," "C8178," "C8179," "C8190," "C8191," "C8192," "C8193," "C8194," "C8195," "C8196," "C8197," "C8198," "C8199," "C8200," "C8201," "C8202," "C8203," "C8204," "C8205," "C8206," "C8207," "C8208," "C8209," "C8210," "C8211," "C8212," "C8213," "C8214," "C8215," "C8216," "C8217," "C8218," "C8219," "C8220," "C8221," "C8222," "C8223," "C8224," "C8225," "C8226," "C8227," "C8228," "C8229," "C8230," "C8231," "C8232," "C8233," "C8234," "C8235," "C8236," "C8237," "C8238," "C8239," "C8240," "C8241," "C8242," "C8243," "C8244," "C8245," "C8246," "C8247," "C8248," "C8249," "C8250," "C8251," "C8252," "C8253," "C8254," "C8255," "C8256," "C8257," "C8258," "C8259," "C8260," "C8261," "C8262," "C8263," "C8264," "C8265," "C8266," "C8267," "C8268," "C8269," "C8280," "C8281," "C8282," "C8283," "C8284," "C8285," "C8286," "C8287," "C8288," "C8289," "C8290," "C8291," "C8292," "C8293," "C8294," "C8295," "C8296," "C8297," "C8298," "C8299," "C8300," "C8301," "C8302," "C8303," "C8304," "C8305," "C8306," "C8307," "C8308," "C8309," "C8310," "C8311," "C8312," "C8313," "C8314," "C8315," "C8316," "C8317," "C8318," "C8319," "C8330," "C8331," "C8332," "C8333," "C8334," "C8335," "C8336," "C8337," "C8338," "C8339," "C8350," "C8351," "C8352," "C8353," "C8354," "C8355," "C8356," "C8357," "C8358," "C8359," "C8370," "C8371," "C8372," "C8373," "C8374," "C8375," "C8376," "C8377," "C8378," "C8379," "C8380," "C8381," "C8382," "C8383," "C8384," "C8385," "C8386," "C8387," "C8388," "C8389," "C8390," "C8391," "C8392," "C8393," "C8394," "C8395," "C8396," "C8397," "C8398," "C8399," "C8400," "C8401," "C8402," "C8403," "C8404," "C8405," "C8406," "C8407," "C8408," "C8409," "C8410," "C8411," "C8412," "C8413," "C8414," "C8415," "C8416," "C8417," "C8418," "C8419," "C8440," "C8441," "C8442," "C8443," "C8444," "C8445," "C8446," "C8447," "C8448," "C8449," "C8460," "C8461," "C8462," "C8463," "C8464," "C8465," "C8466," "C8467," "C8468," "C8469," "C8470," "C8471," "C8472," "C8473," "C8474," "C8475," "C8476," "C8477," "C8478," "C8479," "C8490," "C8491," "C8492," "C8493," "C8494," "C8495," "C8496," "C8497," "C8498," "C8499," "C84A0," "C84A1," "C84A2," "C84A3," "C84A4," "C84A5," "C84A6," "C84A7," "C84A8," "C84A9," "C84Z0," "C84Z1," "C84Z2," "C84Z3," "C84Z4," "C84Z5," "C84Z6," "C84Z7," "C84Z8," "C84Z9," "C8510," "C8511," "C8512," "C8513," "C8514," "C8515," "C8516," "C8517," "C8518," "C8519," "C8520," "C8521," "C8522," "C8523," "C8524," "C8525," "C8526," "C8527," "C8528," "C8529," "C8580," "C8581," "C8582," "C8583," "C8584," "C8585," "C8586," "C8587," "C8588," "C8589," "C8590," "C8591," "C8592," "C8593," "C8594," "C8595," "C8596," "C8597," "C8598," "C8599," "C860," "C861," "C862," "C863," "C864," "C865," "C866," "C880," "C882," "C883," "C884," "C888," "C889," "C9000," "C9001," "C9002," "C9020," "C9021," "C9022," "C9030," "C9031," "C9032," "C960," "C962," "C9620," "C9621," "C9622," "C9629," "C964," "C969," "C96A," "C96Z," "D47Z9"
Solid cancers	"C000," "C001," "C002," "C003," "C004," "C005," "C006," "C008," "C009," "C01," "C020," "C021," "C022," "C023," "C024," "C028," "C029," "C030," "C031," "C039," "C040," "C041," "C048," "C049," "C050," "C051," "C052," "C058," "C059," "C060," "C061," "C062," "C0680," "C0689," "C069," "C07," "C080," "C081," "C089," "C090," "C091," "C098," "C099," "C100," "C101," "C102," "C103," "C104," "C108," "C109," "C110," "C111," "C112," "C113," "C118," "C119," "C12," "C130," "C131," "C132," "C138," "C139," "C140," "C142," "C148," "C153," "C154," "C155," "C158," "C159," "C160," "C161," "C162," "C163," "C164," "C165," "C166," "C168," "C169," "C170," "C171," "C172," "C173," "C178," "C179," "C180," "C181," "C182," "C183," "C184," "C185," "C186," "C187," "C188," "C189," "C19," "C20," "C210," "C211," "C212," "C218," "C220," "C221," "C222," "C223," "C224," "C227," "C228," "C229," "C23," "C240," "C241," "C248," "C249," "C250," "C251," "C252," "C253," "C254," "C257," "C258," "C259," "C260," "C261," "C269," "C300," "C301," "C310," "C311," "C312," "C313," "C318," "C319," "C320," "C321," "C322," "C323," "C328," "C329," "C33," "C3400," "C3401," "C3402," "C3410," "C3411," "C3412," "C342," "C3430," "C3431," "C3432," "C3480," "C3481," "C3482," "C3490," "C3491," "C3492," "C37," "C380," "C381," "C382," "C383," "C384," "C388," "C390," "C399," "C4000," "C4001," "C4002," "C4010," "C4011," "C4012," "C4020," "C4021," "C4022," "C4030," "C4031," "C4032," "C4080," "C4081," "C4082," "C4090," "C4091," "C4092," "C410," "C411," "C412," "C413," "C414," "C419," "C430," "C4310," "C4311," "C43111," "C43112," "C4312," "C43121," "C43122," "C4320," "C4321," "C4322," "C4330," "C4331," "C4339," "C434," "C4351," "C4352," "C4359," "C4360," "C4361," "C4362," "C4370," "C4371," "C4372," "C438," "C439," "C4400," "C4409," "C44101," "C44102," "C441021," "C441022," "C44109," "C441091," "C441092," "C44131," "C441321," "C441322," "C441391," "C441392," "C44191," "C44192," "C441921," "C441922," "C44199," "C441991," "C441992," "C44201," "C44202," "C44209," "C44291," "C44292," "C44299," "C44300," "C44301," "C44309," "C44390," "C44391," "C44399," "C4440," "C4449," "C44500," "C44501," "C44509," "C44590," "C44591," "C44599," "C44601," "C44602," "C44609," "C44691," "C44692," "C44699," "C44701," "C44702," "C44709," "C44791," "C44792," "C44799," "C4480," "C4489," "C4490," "C4499," "C450," "C451," "C452," "C457," "C459," "C460," "C461," "C462," "C463," "C464,"

(*Continued*)

 © 2023. The Author(s).

Supplementary Table S1 (*Continued*)

	ICD-10 Codes
	"C4650," "C4651," "C4652," "C467," "C469," "C470," "C4710," "C4711," "C4712," "C4720," "C4721," "C4722," "C473," "C474," "C475," "C476," "C478," "C479," "C480," "C481," "C482," "C488," "C490," "C4910," "C4911," "C4912," "C4920," "C4921," "C4922," "C493," "C494," "C495," "C496," "C498," "C499," "C49A0," "C49A1," "C49A2," "C49A3," "C49A4," "C49A5," "C49A9," "C4A0," "C4A10," "C4A11," "C4A111," "C4A112," "C4A12," "C4A121," "C4A122," "C4A20," "C4A21," "C4A22," "C4A30," "C4A31," "C4A39," "C4A4," "C4A51," "C4A52," "C4A59," "C4A60," "C4A61," "C4A62," "C4A70," "C4A71," "C4A72," "C4A8," "C4A9," "C50011," "C50012," "C50019," "C50021," "C50022," "C50029," "C50111," "C50112," "C50119," "C50121," "C50122," "C50129," "C50211," "C50212," "C50219," "C50221," "C50222," "C50229," "C50311," "C50312," "C50319," "C50321," "C50322," "C50329," "C50411," "C50412," "C50419," "C50421," "C50422," "C50429," "C50511," "C50512," "C50519," "C50521," "C50522," "C50529," "C50611," "C50612," "C50619," "C50621," "C50622," "C50629," "C50811," "C50812," "C50819," "C50821," "C50822," "C50829," "C50911," "C50912," "C50919," "C50921," "C50922," "C50929," "C510," "C511," "C512," "C518," "C519," "C52," "C530," "C531," "C538," "C539," "C540," "C541," "C542," "C543," "C548," "C549," "C55," "C561," "C562," "C569," "C5700," "C5701," "C5702," "C5710," "C5711," "C5712," "C5720," "C5721," "C5722," "C573," "C574," "C577," "C578," "C579," "C58," "C600," "C601," "C602," "C608," "C609," "C61," "C6200," "C6201," "C6202," "C6210," "C6211," "C6212," "C6290," "C6291," "C6292," "C6300," "C6301," "C6302," "C6310," "C6311," "C6312," "C632," "C637," "C638," "C639," "C641," "C642," "C649," "C651," "C652," "C659," "C661," "C662," "C669," "C670," "C671," "C672," "C673," "C674," "C675," "C676," "C677," "C678," "C679," "C680," "C681," "C688," "C689," "C6900," "C6901," "C6902," "C6910," "C6911," "C6912," "C6920," "C6921," "C6922," "C6930," "C6931," "C6932," "C6940," "C6941," "C6942," "C6950," "C6951," "C6952," "C6960," "C6961," "C6962," "C6980," "C6981," "C6982," "C6990," "C6991," "C6992," "C700," "C701," "C709," "C710," "C711," "C712," "C713," "C714," "C715," "C716," "C717," "C718," "C719," "C720," "C721," "C7220," "C7221," "C7222," "C7230," "C7231," "C7232," "C7240," "C7241," "C7242," "C7250," "C7259," "C729," "C73," "C7400," "C7401," "C7402," "C7410," "C7411," "C7412," "C7490," "C7491," "C7492," "C750," "C751," "C752," "C753," "C754," "C755," "C758," "C759," "C760," "C761," "C762," "C763," "C7640," "C7641," "C7642," "C7650," "C7651," "C7652," "C768," "C7A00," "C7A010," "C7A011," "C7A012," "C7A019," "C7A020," "C7A021," "C7A022," "C7A023," "C7A024," "C7A025," "C7A026," "C7A029," "C7A090," "C7A091," "C7A092," "C7A093," "C7A094," "C7A095," "C7A096," "C7A098," "C7A1," "C7A8," "D469," "E3121," "E3122," "E3123"

Supplementary Table S2 ICD-10 codes for the comorbidities used in the study (International Classification of Diseases, 10th revision [ICD-10])

	ICD-10 codes
AIDS	"B20," "O98711," "O98712," "O98713," "O98719," "O9872," "O9873," "Z21"
Alcohol abuse	"F1010," "F1011," "F10120," "F10121," "F10129," "F10130," "F10131," "F10132," "F10139," "F1014," "F10150," "F10151," "F10159," "F10180," "F10181," "F10182," "F10188," "F1019," "F1020," "F1021," "F10220," "F10221," "F10229," "F10230," "F10231," "F10232," "F10239," "F1024," "F10250," "F10251," "F10259," "F1026," "F1027," "F10280," "F10281," "F10282," "F10288," "F1029," "F1094," "F10950," "F10951," "F10959," "F1096," "F1097," "F10980," "G621," "I426," "K2920," "K2921," "O99310," "O99311," "O99312," "O99313," "O99314," "O99315"
Chronic lung disease	"J410," "J411," "J418," "J42," "J430," "J431," "J432," "J438," "J439," "J440," "J441," "J449," "J4520," "J4521," "J4522," "J4530," "J4531," "J4532," "J4540," "J4541," "J4542," "J4550," "J4551," "J4552," "J45901," "J45902," "J45909," "J45990," "J45991," "J45998," "J470," "J471," "J479," "J60," "J61," "J620," "J628," "J630," "J631," "J632," "J633," "J634," "J635," "J636," "J64," "J65," "J660," "J661," "J662," "J668," "J670," "J671," "J672," "J673," "J674," "J675," "J676," "J677," "J678," "J679," "J684," "J701," "J703"
Dementia	"F0150," "F0151," "F0280," "F0281," "F0390," "F0391," "G300," "G301," "G308," "G309," "G3101," "G3109," "G311," "G312," "G3181," "G3182," "G3183," "G3185," "G3189," "G319"
Depression	"F0631," "F0632," "F0634," "F320," "F321," "F322," "F323," "F328," "F3281," "F3289," "F329," "F330," "F331," "F332," "F333," "F338," "F339," "F341"
Diabetes mellitus	"E0800," "E0801," "E0810," "E0811," "E089," "E0900," "E0901," "E0910," "E0911," "E099," "E1010," "E1011," "E109," "E1100," "E1101," "E1110," "E1111," "E119," "E1300," "E1301," "E1310," "E1311," "E139," "O24011," "O24012," "O24013," "O24019," "O2402," "O2403," "O24111," "O24112," "O24113," "O24119," "O2412," "O2413," "O24311," "O24312," "O24313," "O24319," "O2432," "O2433," "O24410," "O24414," "O24415," "O24419," "O24420," "O24424,"

 © 2023. The Author(s).

Supplementary Table S2 (*Continued*)

	ICD-10 codes
	"O24425," "O24429," "O24430," "O24434," "O24435," "O24439," "O24811," "O24812," "O24813," "O24819," "O2482," "O2483," "O24911," "O24912," "O24913," "O24919," "O2492," "O2493," "E0821," "E0822," "E0829," "E08311," "E08319," "E08321," "E083211," "E083212," "E083213," "E083219," "E08329," "E083291," "E083292," "E083293," "E083299," "E08331," "E083311," "E083312," "E083313," "E083319," "E08339," "E083391," "E083392," "E083393," "E083399," "E08341," "E083411," "E083412," "E083413," "E083419," "E08349," "E083491," "E083492," "E083493," "E083499," "E08351," "E083511," "E083512," "E083513," "E083519," "E083521," "E083522," "E083523," "E083529," "E083531," "E083532," "E083533," "E083539," "E083541," "E083542," "E083543," "E083549," "E083551," "E083552," "E083553," "E083559," "E08359," "E083591," "E083592," "E083593," "E083599," "E0836," "E0837 × 1," "E0837 × 2," "E0837 × 3," "E0837 × 9," "E0839," "E0840," "E0841," "E0842," "E0843," "E0844," "E0849," "E0851," "E0852," "E0859," "E08610," "E08618," "E08620," "E08621," "E08622," "E08628," "E08630," "E08638," "E08641," "E08649," "E0865," "E0869," "E088," "E0921," "E0922," "E0929," "E09311," "E09319," "E09321," "E093211," "E093212," "E093213," "E093219," "E09329," "E093291," "E093292," "E093293," "E093299," "E09331," "E093311," "E093312," "E093313," "E093319," "E09339," "E093391," "E093392," "E093393," "E093399," "E09341," "E093411," "E093412," "E093413," "E093419," "E09349," "E093491," "E093492," "E093493," "E093499," "E09351," "E093511," "E093512," "E093513," "E093519," "E093521," "E093522," "E093523," "E093529," "E093531," "E093532," "E093533," "E093539," "E093541," "E093542," "E093543," "E093549," "E093551," "E093552," "E093553," "E093559," "E09359," "E093591," "E093592," "E093593," "E093599," "E0936," "E0937 × 1," "E0937 × 2," "E0937 × 3," "E0937 × 9," "E0939," "E0940," "E0941," "E0942," "E0943," "E0944," "E0949," "E0951," "E0952," "E0959," "E09610," "E09618," "E09620," "E09621," "E09622," "E09628," "E09630," "E09638," "E09641," "E09649," "E0965," "E0969," "E098," "E1021," "E1022," "E1029," "E10311," "E10319," "E10321," "E103211," "E103212," "E103213," "E103219," "E10329," "E103291," "E103292," "E103293," "E103299," "E10331," "E103311," "E103312," "E103313," "E103319," "E10339," "E103391," "E103392," "E103393," "E103399," "E10341," "E103411," "E103412," "E103413," "E103419," "E10349," "E103491," "E103492," "E103493," "E103499," "E10351," "E103511," "E103512," "E103513," "E103519," "E103521," "E103522," "E103523," "E103529," "E103531," "E103532," "E103533," "E103539," "E103541," "E103542," "E103543," "E103549," "E103551," "E103552," "E103553," "E103559," "E10359," "E103591," "E103592," "E103593," "E103599," "E1036," "E1037 × 1," "E1037 × 2," "E1037 × 3," "E1037 × 9," "E1039," "E1040," "E1041," "E1042," "E1043," "E1044," "E1049," "E1051," "E1052," "E1059," "E10610," "E10618," "E10620," "E10621," "E10622," "E10628," "E10630," "E10638," "E10641," "E10649," "E1065," "E1069," "E108," "E1121," "E1122," "E1129," "E11311," "E11319," "E11321," "E113211," "E113212," "E113213," "E113219," "E11329," "E113291," "E113292," "E113293," "E113299," "E11331," "E113311," "E113312," "E113313," "E113319," "E11339," "E113391," "E113392," "E113393," "E113399," "E11341," "E113411," "E113412," "E113413," "E113419," "E11349," "E113491," "E113492," "E113493," "E113499," "E11351," "E113511," "E113512," "E113513," "E113519," "E113521," "E113522," "E113523," "E113529," "E113531," "E113532," "E113533," "E113539," "E113541," "E113542," "E113543," "E113549," "E113551," "E113552," "E113553," "E113559," "E11359," "E113591," "E113592," "E113593," "E113599," "E1136," "E1137 × 1," "E1137 × 2," "E1137 × 3," "E1137 × 9," "E1139," "E1140," "E1141," "E1142," "E1143," "E1144," "E1149," "E1151," "E1152," "E1159," "E11610," "E11618," "E11620," "E11621," "E11622," "E11628," "E11630," "E11638," "E11641," "E11649," "E1165," "E1169," "E118," "E1321," "E1322," "E1329," "E13311," "E13319," "E13321," "E133211," "E133212," "E133213," "E133219," "E13329," "E133291," "E133292," "E133293," "E133299," "E13331," "E133311," "E133312," "E133313," "E133319," "E13339," "E133391," "E133392," "E133393," "E133399," "E13341," "E133411," "E133412," "E133413," "E133419," "E13349," "E133491," "E133492," "E133493," "E133499," "E13351," "E133511," "E133512," "E133513," "E133519," "E133521," "E133522," "E133523," "E133529," "E133531," "E133532," "E133533," "E133539," "E133541," "E133542," "E133543," "E133549," "E133551," "E133552," "E133553," "E133559," "E13359," "E133591," "E133592," "E133593," "E133599," "E1336," "E1337 × 1," "E1337 × 2," "E1337 × 3," "E1337 × 9," "E1339," "E1340," "E1341," "E1342," "E1343," "E1344," "E1349," "E1351," "E1352," "E1359," "E13610," "E13618," "E13620," "E13621," "E13622," "E13628," "E13630," "E13638," "E13641," "E13649," "E1365," "E1369," "E138"
Drug abuse	"F1110," "F1111," "F11120," "F11121," "F11122," "F11129," "F1113," "F1114," "F11181," "F11182," "F11188," "F1119," "F1120," "F1121," "F11220," "F11221," "F11222," "F11229," "F1123," "F1124," "F11281," "F11282," "F11288," "F1129," "F1210," "F1211," "F12120," "F12121," "F12122," "F12129," "F1213," "F12180," "F12188," "F1219," "F1220," "F1221," "F12220," "F12221," "F12222," "F12229," "F1223," "F12280," "F12288," "F1229," "F1310," "F1311," "F13120," "F13121," "F13129," "F13130," "F13131," "F13132," "F13139," "F1314," "F13180," "F13181," "F13182," "F13188," "F1319," "F1320," "F1321," "F13220," "F13221," "F13229," "F13230," "F13231," "F13232," "F13239," "F1324," "F1326," "F1327," "F13280," "F13281,"

(*Continued*)

 © 2023. The Author(s).

Supplementary Table S2 (*Continued*)

	ICD-10 codes
	“F13282,” “F13288,” “F1329,” “F1410,” “F1411,” “F14120,” “F14121,” “F14122,” “F14129,” “F1413,” “F1414,” “F14180,” “F14181,” “F14182,” “F14188,” “F1419,” “F1420,” “F1421,” “F14220,” “F14221,” “F14222,” “F14229,” “F1423,” “F1424,” “F14280,” “F14281,” “F14282,” “F14288,” “F1429,” “F1510,” “F1511,” “F15120,” “F15121,” “F15122,” “F15129,” “F1513,” “F1514,” “F15180,” “F15181,” “F15182,” “F15188,” “F1519,” “F1520,” “F1521,” “F15220,” “F15221,” “F15222,” “F15229,” “F1523,” “F1524,” “F15280,” “F15281,” “F15282,” “F15288,” “F1529,” “F1610,” “F1611,” “F16120,” “F16121,” “F16122,” “F16129,” “F1614,” “F16180,” “F16183,” “F16188,” “F1619,” “F1620,” “F1621,” “F16220,” “F16221,” “F16229,” “F1624,” “F16280,” “F16283,” “F16288,” “F1629,” “F1810,” “F1811,” “F18120,” “F18121,” “F18129,” “F1814,” “F1817,” “F18180,” “F18188,” “F1819,” “F1820,” “F1821,” “F18220,” “F18221,” “F18229,” “F1824,” “F1827,” “F18280,” “F18288,” “F1829,” “F1910,” “F1911,” “F19120,” “F19121,” “F19122,” “F19129,” “F19130,” “F19131,” “F19132,” “F19139,” “F1914,” “F1916,” “F1917,” “F19180,” “F19181,” “F19182,” “F19188,” “F1919,” “F1920,” “F1921,” “F19220,” “F19221,” “F19222,” “F19229,” “F19230,” “F19231,” “F19232,” “F19239,” “F1924,” “F1926,” “F1927,” “F19280,” “F19281,” “F19282,” “F19288,” “F1929,” “O99320,” “O99321,” “O99322,” “O99323,” ”O99324,” “O99325”
Hypertension	“I10,” “I160,” “I169,” “O10011,” “O10012,” “O10013,” “O10019,” “O1002,” “O1003,” “H35031,” “H35032,” “H35033,” “H35039,” “I119,” “I129,” “I1310,” “I150,” “I151,” “I152,” “I158,” “I159,” “I161,” “I674,” “O10111,” “O10112,” “O10113,” “O10119,” “O1012,” “O1013,” “O10211,” “O10212,” “O10213,” “O10219,” “O1022,” “O1023,” “O10311,” “O10312,” “O10313,” “O10319,” “O1032,” “O1033,” “O10411,” “O10412,” “O10413,” “O10419,” “O1042,” “O1043,” “O10911,” “O10912,” “O10913,” “O10919,” “O1092,” “O1093,” “O111,” “O112,” “O113,” “O114,” “O115,” “O119,” “O161,” “O162,” “O163,” ”O164,” “O165,” “O169”
Hypothyroidism	“E000,” “E001,” “E002,” “E009,” “E010,” “E011,” “E012,” “E018,” “E02,” “E030,” “E031,” “E032,” “E033,” “E034,” “E035,” “E038,” “E039,” “E890”
Obesity	“E6601,” “E6609,” “E661,” “E662,” “E668,” “E669,” “O99210,” “O99211,” “O99212,” “O99213,” “O99214,” “O99215,” “R939,” “Z6830,” “Z6831,” “Z6832,” “Z6833,” “Z6834,” “Z6835,” “Z6836,” “Z6837,” “Z6838,” “Z6839,” “Z6841,” “Z6842,” “Z6843,” “Z6844,” “Z6845,” “Z6854”
PVD	“A5200,” “A5201,” “A5202,” “A5209,” “I700,” “I701,” “I70201,” “I70202,” “I70203,” “I70208,” “I70209,” “I70211,” “I70212,” “I70213,” “I70218,” “I70219,” “I70221,” “I70222,” “I70223,” “I70228,” “I70229,” “I70231,” “I70232,” “I70233,” “I70234,” “I70235,” “I70238,” “I70239,” “I70241,” “I70242,” “I70243,” “I70244,” “I70245,” “I70248,” “I70249,” “I7025,” “I70261,” “I70262,” “I70263,” “I70268,” “I70269,” “I70291,” “I70292,” “I70293,” “I70298,” “I70299,” “I70301,” “I70302,” “I70303,” “I70308,” “I70309,” “I70311,” “I70312,” “I70313,” “I70318,” “I70319,” “I70321,” “I70322,” “I70323,” “I70328,” “I70329,” “I70331,” “I70332,” “I70333,” “I70334,” “I70335,” “I70338,” “I70339,” “I70341,” “I70342,” “I70343,” “I70344,” “I70345,” “I70348,” “I70349,” “I7035,” “I70361,” “I70362,” “I70363,” “I70368,” “I70369,” “I70391,” “I70392,” “I70393,” “I70398,” “I70399,” “I70401,” “I70402,” “I70403,” “I70408,” “I70409,” “I70411,” “I70412,” “I70413,” “I70418,” “I70419,” “I70421,” “I70422,” “I70423,” “I70428,” “I70429,” “I70431,” “I70432,” “I70433,” “I70434,” “I70435,” “I70438,” “I70439,” “I70441,” “I70442,” “I70443,” “I70444,” “I70445,” “I70448,” “I70449,” “I7045,” “I70461,” “I70462,” “I70463,” “I70468,” “I70469,” “I70491,” “I70492,” “I70493,” “I70498,” “I70499,” “I70501,” “I70502,” “I70503,” “I70508,” “I70509,” “I70511,” “I70512,” “I70513,” “I70518,” “I70519,” “I70521,” “I70522,” “I70523,” “I70528,” “I70529,” “I70531,” “I70532,” “I70533,” “I70534,” “I70535,” “I70538,” “I70539,” “I70541,” “I70542,” “I70543,” “I70544,” “I70545,” “I70548,” “I70549,” “I7055,” “I70561,” “I70562,” “I70563,” “I70568,” “I70569,” “I70591,” “I70592,” “I70593,” “I70598,” “I70599,” “I70601,” “I70602,” “I70603,” “I70608,” “I70609,” “I70611,” “I70612,” “I70613,” “I70618,” “I70619,” “I70621,” “I70622,” “I70623,” “I70628,” “I70629,” “I70631,” “I70632,” “I70633,” “I70634,” “I70635,” “I70638,” “I70639,” “I70641,” “I70642,” “I70643,” “I70644,” “I70645,” “I70648,” “I70649,” “I7065,” “I70661,” “I70662,” “I70663,” “I70668,” “I70669,” “I70691,” “I70692,” “I70693,” “I70698,” “I70699,” “I70701,” “I70702,” “I70703,” “I70708,” “I70709,” “I70711,” “I70712,” “I70713,” “I70718,” “I70719,” “I70721,” “I70722,” “I70723,” “I70728,” “I70729,” “I70731,” “I70732,” “I70733,” “I70734,” “I70735,” “I70738,” “I70739,” “I70741,” “I70742,” “I70743,” “I70744,” “I70745,” “I70748,” “I70749,” “I7075,” “I70761,” “I70762,” “I70763,” “I70768,” “I70769,” “I70791,” “I70792,” “I70793,” “I70798,” “I70799,” “I708,” “I7090,” “I7091,” “I7092,” “I7100,” “I7101,” “I7102,” “I7103,” “I711,” “I712,” “I713,” “I714,” “I715,” “I716,” “I718,” “I719,” “I720,” “I721,” “I722,” “I723,” “I724,” “I725,” “I726,” “I728,” “I729,” “I7301,” “I731,” “I7381,” “I7389,” “I739,” “I7401,” “I7409,” “I7410,” “I7411,” “I7419,” “I742,” “I743,” “I744,” “I745,” “I748,” “I749,” “I75011,” “I75012,” “I75013,” “I75019,” “I75021,” “I75022,” “I75023,” “I75029,” “I7581,” “I7589,” “I770,” “I771,” “I772,” “I773,” “I774,” “I775,” “I776,” “I7770,” “I7771,” “I7772,” “I7773,” “I7774,” “I7775,” “I7776,” “I7777,” “I7779,” “I77810,” “I77811,” “I77812,” “I77819,” “I7789,” “I779,” “I780,” “I781,” “I788,” “I789,” “I790,”

 © 2023. The Author(s).

Supplementary Table S2 (*Continued*)

	ICD-10 codes
	"I791," "I798," "I998," "I999," "K31811," "K31819," "K551," "K558," "K559," "Z95820," "Z95828"
Deficiency anemia	"D501," "D508," "D509," "D510," "D511," "D512," "D513," "D518," "D519," "D520," "D521," "D528," "D529," "D530," "D531," "D532," "D538," "D539," "D630," "D631," "D638," "D649," "O99011," "O99012," "O99013," "O99019"
Blood loss	"D500," "O9081," "O9902," "O9903"
Coagulopathy	"D6109," "D611," "D612," "D613," "D61810," "D61811," "D61818," "D6182," "D6189," "D619," "D65," "D66," "D67," "D680," "D681," "D682," "D68311," "D68312," "D68318," "D6832," "D684," "D688," "D689," "D691," "D693," "D6941," "D6942," "D6949," "D6951," "D6959," "D696," "D698," "D699," "D7582," "O99111," "O99112," "O99113," "O99119," "O9912," "O9913"
Pulmonary HTN	"I270," "I271," "I272," "I2720," "I2721," "I2722," "I2723," "I2724," "I2729," "I2781," "I2782," "I2783," "I2789," "I279," "I280," "I281," "I288," "I289"
PUD	"K250," "K251," "K252," "K253," "K254," "K255," "K256," "K257," "K259," "K260," "K261," "K262," "K263," "K264," "K265," "K266," "K267," "K269," "K270," "K271," "K272," "K273," "K274," "K275," "K276," "K277," "K279," "K280," "K281," "K282," "K283," "K284," "K285," "K286," "K287," "K289"
Weight loss	"E40," "E41," "E42," "E43," "E440," "E441," "E45," "E46," "E640," "O2510," "O2511," "O2512," "O2513," "O252," "O253," "R634," "R64"
Valvular disease	"A1884," "A3282," "A3951," "A5203," "B3321," "B376," "I011," "I018," "I019," "I020," "I050," "I051," "I052," "I058," "I059," "I060," "I061," "I062," "I068," "I069," "I070," "I071," "I072," "I078," "I079," "I080," "I081," "I082," "I083," "I088," "I089," "I091," "I0989," "I330," "I339," "I340," "I341," "I342," "I348," "I349," "I350," "I351," "I352," "I358," "I359," "I360," "I361," "I362," "I368," "I369," "I370," "I371," "I372," "I378," "I379," "I38," "I39," "M3211," "Q220," "Q221," "Q222," "Q223," "Q224," "Q225," "Q226," "Q228," "Q229," "Q230," "Q231," "Q232," "Q233," "Q234," "Q238," "Q239," "T8201XA," "T8201XD," "T8201XS," "T8202XA," "T8202XD," "T8202XS," "T8203XA," "T8203XD," "T8203XS," "T8209XA," "T8209XD," "T8209XS," "T82221A," "T82221D," "T82221S," "T82222A," "T82222D," "T82222S," "T82223A," "T82223D," "T82223S," "T82228A," "T82228D," "T82228S," "T826XXA," "T826XXD," "T826XXS," "Z952," "Z953," "Z954"

Supplementary Table S3 ICD-10 codes for outcomes and procedures used in study design (International Classification of Diseases, 10th revision [ICD-10])

	ICD-10 codes
Cardiogenic shock	"R570," "T8111XA," "T8111XD," "T8111," "T8111XS"
Total SCA	
Not procedure-related arrest	"I462," "I468," "I469"
Procedure-related arrest	"I97120," "I97121," "I97710," "I97711"
VF	"I490," "I4901," "I4902"
Cardiac MRI	"B236Y0Z," "B236YZZ," "B236ZZZ"
Right HC	"4A023N6"
Left HC	"4A023N7"
Combined HC	"4A023N8"
IABP	"5A02110," "5A02115," "5A02210," "5A02215"
VA-ECMO	"5A15223," "5A1522F," "5A1522G," "5A15A2G"
PVAD	"5A02116," "5A0211D," "5A02216," "5A0221D"
LVAD	"02HA0QZ," "02HA3QZ," "02HA4QZ"
Heart transplant	"02YA0Z0," "02YA0Z1," "02YA0Z2"

 © 2023. The Author(s).
